# Occurrence of Microplastic Pollution at Oyster Reefs and Other Coastal Sites in the Mississippi Sound, USA: Impacts of Freshwater Inflows from Flooding

**DOI:** 10.3390/toxics8020035

**Published:** 2020-05-15

**Authors:** Austin Scircle, James V. Cizdziel, Louis Tisinger, Tarun Anumol, Darren Robey

**Affiliations:** 1 Department of Chemistry and Biochemistry, University of Mississippi, University, MS 38677, USA; arscircl@go.olemiss.edu; 2 Agilent Technologies Inc., 2850 Centerville Road, Wilmington, DE 19808, USA; louis.tisinger@agilent.com (L.T.); tarun.anumol@agilent.com (T.A.); darren.robey@agilent.com (D.R.)

**Keywords:** plastic pollution, oysters, Mississippi Sound, fluorescence microscopy, laser direct infrared analysis, LDIR, bulk water sampling, Bonnet Carré Spillway

## Abstract

Much of the seafood that humans consume comes from estuaries and coastal areas where microplastics (MPs) accumulate, due in part to continual input and degradation of plastic litter from rivers and runoff. As filter feeders, oysters (*Crassostrea virginica*) are especially vulnerable to MP pollution. In this study, we assessed MP pollution in water at oyster reefs along the Mississippi Gulf Coast when: (1) historic flooding of the Mississippi River caused the Bonnet Carré Spillway to remain open for a record period of time causing major freshwater intrusion to the area and deleterious impacts on the species and (2) the spillway was closed, and normal salinity conditions resumed. Microplastics (~25 µm–5 mm) were isolated using a single-pot method, preparing samples in the same vessel (Mason jars) used for their collection right up until the MPs were transferred onto filters for analyses. The MPs were quantified using Nile Red fluorescence detection and identified using laser direct infrared (LDIR) analysis. Concentrations ranged from ~12 to 381 particles/L and tended to decrease at sites impacted by major freshwater intrusion. With the spillway open, average MP concentrations were positively correlated with salinity (*r* = 0.87, *p* = 0.05) for sites with three or more samples examined. However, the dilution effect on MP abundances was temporary, and oyster yields suffered from the extended periods of lower salinity. There were no significant changes in the relative distribution of MPs during freshwater intrusions; most of the MPs (>50%) were in the lower size fraction (~25–90 µm) and consisted mostly of fragments (~84%), followed by fibers (~11%) and beads (~5%). The most prevalent plastic was polyester, followed by acrylates/polyurethanes, polyamide, polypropylene, polyethylene, and polyacetal. Overall, this work provides much-needed empirical data on the abundances, morphologies, and types of MPs that oysters are exposed to in the Mississippi Sound, although how much of these MPs are ingested and their impacts on the organisms deserves further scrutiny. This paper is believed to be the first major application of LDIR to the analysis of MPs in natural waters.

## 1. Introduction 

The occurrence of microplastics (MPs) in the aquatic environment is well documented and is the subject of increasing governmental and public attention [1,2,3]. Microplastics have been detected in practically all water bodies that have been studied, including the Arctic Ocean and remote mountain lakes [4,5]. Owing to their small size and pervasiveness in marine waters, MPs can pose a serious threat to certain aquatic organisms, particularly filter-feeding organisms such as mollusks and oysters [2]. Further, the majority of seafood comes from estuaries and coastal areas where MPs accumulate, due to continual input and degradation of plastic litter from rivers and runoff [1]. 

Several studies have shown that MPs can interfere with nutritional uptake, reproduction, and offspring performance in oysters and mussels and influence larval fish ecology [6,7,8,9], but another study has shown that while MPs are readily ingested by oyster larvae, exposure to plastic concentrations exceeding those observed in the environment resulted in no measurable effects on the development or feeding capacity of the larvae over the duration of the study [10]. In any case, MPs are largely regarded not only as an emerging threat to these susceptible species, but also as an additional stressor to marine ecosystems as a whole [11]. Oyster populations have declined due to a variety of converging factors including oil spills, anoxic and freshwater influx events, disease, invasive species, and overfishing, which have culminated in the loss of ~85% of oyster reefs globally and in the functional extinction of oyster populations in areas where oysters once flourished [12]. Given the precarious state of oyster populations, toxicological studies on the effects MPs have on oysters desperately need field studies to provide critical information on the quantity, sizes, and types of MPs found at oyster reefs. However, such empirical data are generally lacking.

There are a few papers on MPs in oysters and at oyster reefs. Li et al. [13] examined MPs in oysters along the Pearl River Estuary in China, finding 1.5 to 7.2 items per gram of tissue (wet weight), which was positively correlated with concentrations in the surrounding water. Keisling et al. [14] found an average of 0.72 MPs per individual oyster (0.18 particles/g). Rochman et al. [15] found plastic debris and fibers > 500 µm in size in 33% of oysters sold for human consumption. Most such studies rely on visual inspection, and undoubtedly many smaller particles go undetected. Thus, MPs are a potential and poorly understood threat to oyster populations and nearby human communities, like those along the Gulf Coast, where oyster farming is a vital part of the economy. Moreover, the levels of MPs in the northern Gulf of Mexico (nGoM) are among the highest reported globally, both on beaches [16] and in the water, where recent surveys along the Louisiana coast measured as much as 150,000 MPs/m^3^ [17]. 

We previously reported on MP pollution in the Mississippi River basin using a novel single-pot method for sample preparation and Nile Red fluorescence for detection and quantitation [18]. Here, we use the same approach to, for the first time, quantify the abundances and morphology of MPs in water at four different oyster reefs and six other sites in the Mississippi Sound located in the nGoM. We also used LDIR analysis to identify the major types of plastics in a subset of the samples. LDIR uses the long-established infrared spectroscopic technique that has been updated and automated in the Agilent’s 8700 system. To the best of our knowledge, this is the first major application of LDIR analyses for the determination, characterization, and identification of MPs in natural waters. Grab samples were collected seasonally for a year, with spring and summer samples taken when historic flooding of the Mississippi River resulted in major freshwater influxes into the western portion of the Mississippi Sound that devastated oyster reefs and with fall and winter samples collected under more normal salinity conditions. Thus, this work also represents a side-by-side comparison of MPs in the Mississippi Sound during a period of intense freshwater intrusion and during more stable salinity conditions. 

## 2. Materials and Methods 

### 2.1. Study Site 

**Figure 1:**
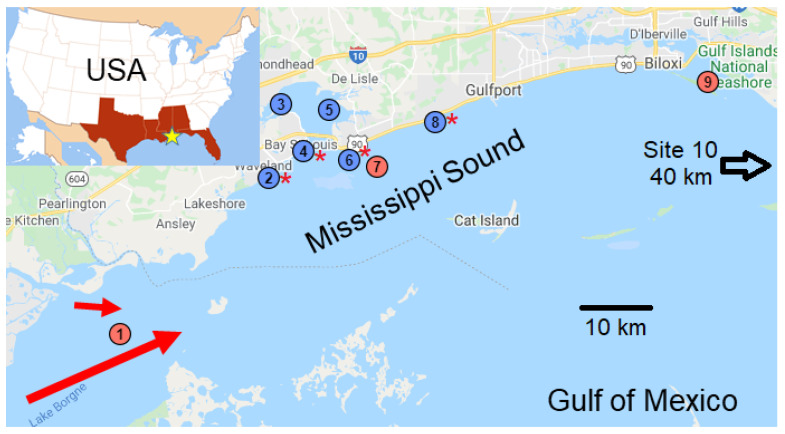
Map showing the sampling locations (circles) in the Mississippi Sound with direction of freshwater inflows from the Bonnet Carré Spillway and the Pearl River (red arrows). Sample site numbers increase from west to east. Sampling sites 2, 4, 6, and 8 (red stars) were at oyster reefs. Red circles represent sites where samples were collected by the Mississippi Department of Environmental Quality, and blue circles sites where samples were collected by the University of Mississippi. Site 10 is off the map, about 40 km to the east in Alabama waters. The inset shows the general study location within the USA.

The Mississippi Sound is a 145 km sound located along the coasts of Mississippi and Alabama in the northern Gulf of Mexico. It has significant commercial and ecological importance for the area and is known for the harvesting of shellfish. However, oyster populations, particularly in the western portion of the sound, have been curtailed in recent years due to pollution (e.g., oil spills) and weather events, including hurricanes and flooding. In this study, we collected water samples from 10 sites, 4 of which were directly above oyster reefs, with the remainder spread throughout the Mississippi Sound (Figure 1). Specific sampling locations (GPS coordinates) and certain water quality measurements are provided in Table 1. Samples were collected in the spring and summer of 2019 during a period of major (historic) flooding along the Mississippi River, as well as in September 2019 and January and February 2020 during more normal salinity conditions (post-flood). During periods of flooding along the lower Mississippi River, its waters are diverted through the Bonnet Carré Spillway, preventing flooding downstream in the city of New Orleans. These floodwaters spill into Lake Ponchartrain, then Lake Borgne, and from there enter the western end of the Mississippi Sound.

### 2.2. Sampling the Waters of the Mississippi Sound

In four separate sampling campaigns, we obtained bulk water (grab) samples from the Mississippi Sound during periods when the Bonnet Carré Spillway was open and dumping freshwater into the Sound and when it had been closed. Our research group at the University of Mississippi (UM) collected samples during April (open spillway) and September (closed spillway) 2019 from sites 2, 3, 4, 5, 6, 8, and the Mississippi Department of Environmental Quality (MDEQ) obtained samples in July 2019 (open spillway) and January 2020 (closed spillway) from sites 1, 7, 9, and 10 (Figure 1 and Table 1). Grab samples were collected just below the surface into glass Mason (canning) jars (946 mL) and tightly capped using metal lids. MDEQ also collected samples <1 m above the bottom using a 4 L Van Dorn sampler, transferring the water to Mason jars (946 mL) immediately after collection. The samples were placed in coolers and shipped to the laboratory at UM for processing. Mason jars are available from many grocery or hardware stores. We used jars that have a 946 mL capacity, but slightly larger (1 L) jars are readily available outside of the USA. Hereafter, we report abundances of MPs/L, adjusting for differences in volume. 

### 2.3. Sample Preparation Using the Single-Pot Method

Samples were processed at our UM microplastics-dedicated laboratory in the same jar they were collected in, using our “single-pot” method described elsewhere [18]. Briefly, the solid canning jar lids were replaced with lids that had a 57 mm diameter opening cut into them, and between that and the jar was placed a round 84 mm-diameter 200 × 600 mesh (~25 um) screen of Monel, a rust-resistant, nickel–copper alloy (Unique Wire Weaving Co. Inc. Hillside, NJ, USA). The jars were inverted, and a stream of air was applied to break the surface tension and force the water to pass through the screen. The screen was then removed and carefully rinsed back into the Mason jar with ultrapure water (purified and filtered (0.2 µm); Milli-Q, Millipore, Burlington, MA, USA). The volume of water used for rinsing was kept low (<~100 mL) to avoid diluting the reactants in the next step, but sufficient to fully rinse the mesh. We used 20 mL of 30% H_2_O_2_ and 20 mL of 0.05 M Fe(II) solution (Fenton’s reagent) to remove natural organic debris in the samples; the method has shown to be an effective pre-treatment for micro-spectroscopic imaging and it avoids acids and prolonged high temperatures known to damage MPs [19,20,21,22]. The solution was vacuum-filtered onto a 10 µm-pore size, 25 mm polycarbonate track-etched filter (Sterlitech Corp., Kent, WA, USA). Method blanks, consisting of ultra-pure H_2_O, were prepared alongside each batch of samples and were subjected to the full sample preparation scheme (filtering, digestion, and staining).

### 2.4. Enumeration of Microplastics Using Fluorescence Microscopy

We used Nile red dye fluorochrome and fluorescence microscopy to detect and characterize the size distribution and morphology of the MPs as detailed elsewhere [18,21]. Briefly, a few drops of Nile red dye (10 µg/mL in methanol) were placed onto the filters with MPs. The samples were allowed to dry for ~20 min in a clean laminar flow hood. The filters were transferred onto microscope slides, and a coverslip was taped above them to prevent particles from shifting when the slides were handled. The samples were analyzed within a day of staining using a Nikon Ti2 Eclipse Fluorescence Microscope. To detect and count putative MPs, we followed a procedure (delineated elsewhere [18]) which took <30 min for each sample. To summarize, the microscopy consisted of imaging the entire filter and counting fluorescing objects lacking biological features such as cellular structure or striations. The dimensions of the objects were determined using built-in measurement tools in the NIS-Elements application (Nikon Instruments Inc., Melville, NY, USA) used to collect the fluorescence microscopy images. The software gives automated measurement of the dimensions of all counted objects, including diameter, length, width, perimeter, area, and circularity. These measurements were used to sort the objects into one of three categories, i.e., fragments, fibers, or beads. Objects with a length-to-width ratio of 3 or greater were defined as fibers, while objects with 0.90 or greater circularity were defined as beads, and all else were categorized as fragments. Method blanks, which rarely exceeded 1/4 of sample counts, were subtracted from the sample results such that data reported herein are blank-subtracted.

### 2.5. Laser Direct Infrared (LDIR) Analysis of Microplastics 

To identify the major types and size fraction of plastics in a subset of seawater samples, we used the Agilent 8700 LDIR analyzer at Agilent Technologies Application Laboratory in Wood Dale, IL, USA. Because this is the first major application of LDIR for microplastics in natural waters, we will briefly introduce the technique before discussing our specific analytical method. 

At its core, the 8700 LDIR is simply an infrared imaging microscope. The key novel aspect is the light source, a proprietary Quantum Cascade Laser (QCL). The QCL is a semi-conductor-based laser in which electrons tunnel through a series of quantum wells and emit light. In a diode-based laser, electron–hole recombination across semiconductor bandgap emits photons. The photon wavelength is set by the chemistry of the materials used. In a QCL, electrons cascade (tunnel) through a series of quantum wells formed by thin layers of semiconductor material. Photon wavelength is not determined by the semiconductor materials but rather by the thickness and distribution of the semiconductor layers [23]. Thus, a QCL can be rapidly tuned through a wavelength range. In some ways, it can be compared to a monochromator approach, since individual wavelengths are used at each time; however, the QCL operates at speeds and wavelength accuracy orders of magnitude better than the older approaches.

The LDIR analyzer used in this study combines the QCL with a single-point mercury cadmium telluride (MCT) detector (thermometrically cooled) and rapid scanning optics, allowing two useful modes of action. In the first, the LDIR parks the frequency at a single wavelength and scans through the objective as it moves over the sample. At each point (pixel), the information is acquired in as little as 40 µs, allowing fast scanning of large areas, significantly faster than traditional with FTIR spectroscopes. In the second mode, the objective is parked at a single point, while the QCL sweeps through the frequency range. A full spectrum is obtained in <1 s. 

The microplastics analysis presented herein utilized both modes. First, the objective rapidly scanned the sample area at the parked frequency. The analysis area varied depending on how the sample was deposited on the slide, but was typically ~500 mm^2^ (range ~145 mm^2^ to ~1100 mm^2^). In any case, the sweep locates particles in the analysis area and obtains information that can be used to describe the size and shape of the particles. Image analysis then rapidly defines the particles and describes their major axis size, area, and shape. Once located, the LDIR then rapidly and automatically moves around the analysis area of each particle and, using the parked objective/frequency sweep mode, obtains a full spectrum in the mid-IR range (1800 cm^−1^ to 975 cm^−1^), conducts a library search, and provides a match. The library used in this study was created from data derived from external sources to include plastics of many different kinds. The library features were created from a database published earlier [24]. For the smallest particles (<30 µm), the system may determine a need to automatically refocus to obtain an optimum spectrum. In this case, the per particle analysis time may be up to 8 s.

For LDIR analysis, we prepared seawater samples as described before (see Sample preparation using the single-pot method), except we did the final filtering using a wire mesh screen with ~25 µm openings. The screen was then submerged in 50% ethanol in a glass vial and subjected to ultrasonication for ~2 min. The screen was then removed with forceps, and the vial was sealed and shipped overnight to Agilent. There, the samples were deposited on MirrIR low-e Kevley microscope slides. The solvent was allowed to evaporate, leaving behind the MPs adhering to the slide. 

The LDIR analyzer was run in trans-reflectance mode, where the system directs IR laser light through the sample (particle), and the light was then reflected back off the reflective slide through the particle as it exited. As noted, the analyzer first scans the slide to locate each particle using IR light at 1800 cm^−1^, a frequency at which little or no absorption occurs, but the light is scattered when encountering a particle. The system utilizes image analysis techniques to determine the boundary of the particle and hence the dimensions. In addition, a full spectrum of each particle covering the range of the instrument is collected and compared to the spectral library in real time. The particle is identified based on this comparison to a spectral database built into the software. Validation of this method was achieved through the use of a blind sample consisting of a blank spiked with MPs composed of polyethylene, polyamide (nylon), polypropylene, and polystyrene, and each of these types of MPs were identified in the analyses. All data analysis and processing were done in real time using the Agilent Clarity Software. 

## 3. Results and Discussion

We found no significant difference (*t*-test, α = 0.05, unequal variances) between the samples collected just below the surface (*n* = 48) and those collected near the bottom (*n* = 28) during the same sampling event and across all sampling sites. The lack of difference by depth is not surprising, given the shallow waters (often <3 m deep) and mixing by wave action. Hereafter, we will not distinguish samples by depth. 

### 3.1. Spatial and Temporal Trends of MPs in the Mississippi Sound 

Microplastic concentrations from all sites and sampling campaigns are summarized in Table 2. The average concentrations ranged from 30 to 192 particles/L across the sites, with concentrations of individual samples ranging from 12 to 381 particles/L. It is not unusual to observe such a wide range of MP concentrations, especially when counting particles as low as 30 µm in bulk water grab samples. Indeed, the sampling method will affect the counts and distribution of the MPs measured [25]. For example, nets are typically used in investigating large areas, with results being reported in particles/m^3^, whereas bulk water sampling, done here, can provide a snapshot at a given site and is generally reported in particles/L. A major drawback to sampling with a net is that it fails to capture particles smaller than the mesh opening (typically 333 µm), and these smaller particles tend to be the most abundant [26]. Conversely, the probability of capturing larger particles in grab samples is lower compared to trawling with a net. 

Using the average concentration of MPs determined throughout the study (129 ± 93 MPs/L) and the average volume of water that an adult oyster filters daily (~189 L) [27], we estimated oysters may be exposed to nearly 24,000 MPs daily (range ~5600 to ~36,000). We stress that this is only an estimate and that MP concentrations and filtering rates will vary depending on site-specific conditions, oyster species, and other factors. Moreover, whether the MPs are actually entrained in oyster tissues likely depends on their size and morphologies and whether they are ingested or just make contact with the tissues (see microplastic morphologies below). Recent studies on oyster ingestion of MPs have shown that oysters are ingesting and retaining between 0.6 and 16.5 MPs per individual [14,15,28,29,30,31]. These studies also show that oysters nearer urban centers often contained higher concentrations of MPs. Given the prevalence of commercial fishing, oil drilling, and shipping ports in the nGoM, oysters along the Gulf Coast could be accumulating a considerable amount of MPs.

**Figure 2:**
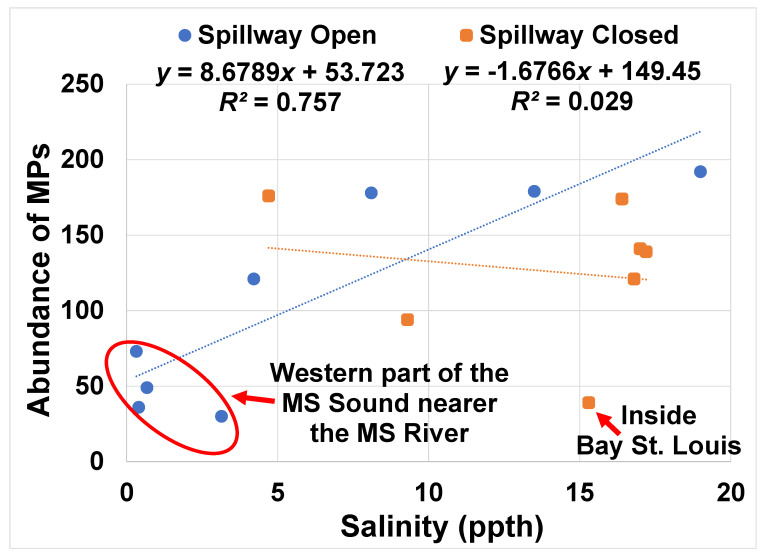
Salinity versus microplastic abundances in the water from sites across the Mississippi (MS) Sound, showing a moderate correlation during freshwater intrusion from flooding (spillway open). All sites were from open water areas in the Gulf, except for Bay St. Louis. Data are means of three or more measurements. ppth = parts per thousand.

Despite the inherent variability in MP concentrations, we observed a moderately positive correlation (*r* = 0.62) between the average MP concentration and salinity at each site (salinity being used as a proxy for lack of freshwater intrusion) when the spillway was open. The correlation (*r*-value) improved to 0.87 after removing data from sites that had only two data points (sites 1 and 5) (Figure 2). There was no such trend when the spillway was closed, suggesting a link between freshwater inflows and MP concentrations, essentially a dilution effect during high river discharges. Indeed, for the majority of the sites, MP concentrations were higher when the spillway was closed (Table 2). Further, the MP concentrations in the seawater were typically higher than what we observed for the Mississippi River and its tributaries [18]. We conclude that seawater along the Mississippi Gulf Coast has higher MP abundances (when including MPs sizes down to ~30 µm) than the river supplying new loads of MPs to the coastal area. This finding that the estuaries have higher concentrations of MPs than their riverine inputs has been observed elsewhere [27]. This is not surprising, as the river is continually flushed of plastics, the estuary serves as a sink for these plastics, and with time the plastics mechanically and photolytically degrade to smaller and smaller particles. 

Because a high degree of spatial variability is common in MP studies [32,33], especially when counting smaller size fractions like in this study, it can be difficult to observe trends, particularly where there are no major point sources. Here, except for during intense freshwater inputs in the western portion of the Sound during the spring and summer, MP concentrations did not show any distinct trend in open water areas based on longitude. However, sites 3 and 5, both located inside Bay St. Louis, tended to have lower concentrations compared to adjacent open waters of the Sound. For example, when the spillway was closed (i.e., “normal” conditions) the average concentration inside the Bay was 54 ± 18 MPs/L (*n* = 5; ±1 SE), while just outside the Bay, the levels were 145 ± 18 MPs/L (*n* = 13; ±1 SE) (see also Table 2). The reason for this is unclear and requires further study, but the circulation, mixing, and flow inside Bay St. Louis are certainly different than those of the other coastal open water sites. Additionally, the bay has its own freshwater input coming from the Wolf River, which could further contribute to the lower concentrations observed. Interestingly, the size distributions of MPs in the samples were generally similar between periods when the spillway was open and when it was closed, with the smallest size fraction, 20–90 μm, being the most common and comprising ~70% of the counted particles.

### 3.2. Microplastic Morphologies

**Figure 3:**
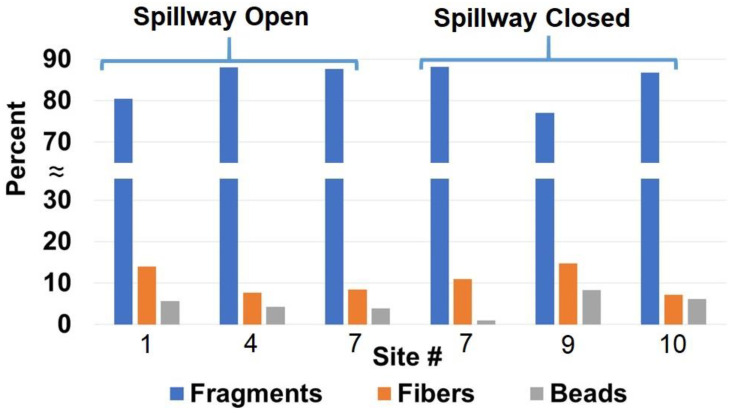
Morphologies of microplastics in water from select sites in the Mississippi Sound. Site number information is given in Table 1.

The microplastic morphologies determined by fluorescence microscopy were generally consistent from site to site (Figure 3). The morphologies were similarly distributed whether the water was gathered in Bay St. Louis or at a reef in the Mississippi Sound. Fragments made up the majority of MPs counted (77–88%), followed by fibers (7–15%), and beads (1–8%). This contrasts with studies that have predominantly found anthropogenic fibers in oyster tissue [14,15,31]. However, most of these studies employed optical microscopy for detection instead of fluorescence microscopy, targeting larger microplastics. A study that used µFT-IR analyses for MP contamination in bivalves found that most of the MPs were indeed fragments [34]. However, it is also possible that fibers may be disproportionally retained because of their shape. Regardless, the connection between levels of MP pollution at reefs and concentrations of MPs ingested by oysters may not exhibit a linear relationship and requires further scrutiny. 

### 3.3. Identification and Quantification of Microplastics Using LDIR

LDIR analysis was applied to identify putative MPs for a selection of samples from sites 1, 7, 9, and 10 (see Figure 1 for locations and Table 3 for LDIR results). The most identified plastic throughout the samples was polyester, followed by acrylates/polyurethanes, and polyamide. Other plastics identified were polypropylene, polyethylene, polyacetal, and polytetrafluoroethylene (PTFE). Whereas polyesters and polyamides are typically used in synthetic fibers, all of the identified plastics are commonly encountered in similar studies [1,2,27,35]. 

**Figure 4:**
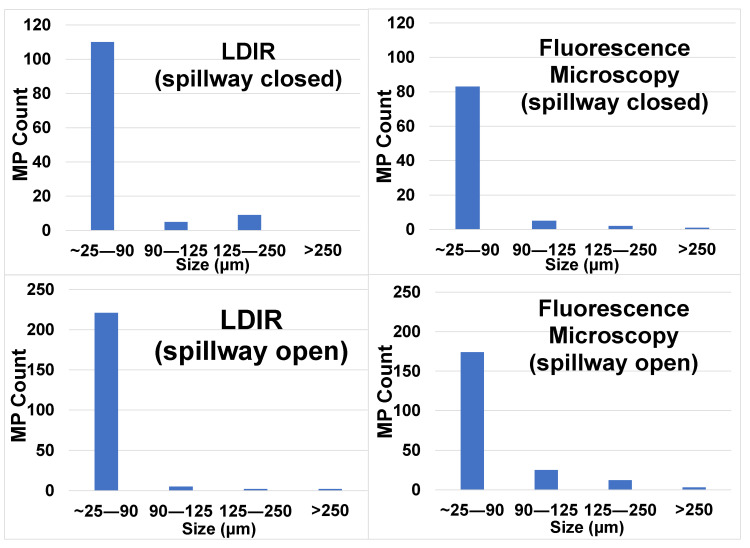
Particle size distribution of microplastics from 1 L of seawater collected in the Mississippi Sound and determined by Laser Direct Infrared (LDIR, **left**) and fluorescence microscopy (**right**). Note the different scale for MP counts for spillway open (**top**, indicating freshwater intrusion) and spillway closed (**bottom**, representing normal salinity conditions).

LDIR data was also used to quantify the abundance of MPs (see MP counts in Table 3, third column) and their size distribution in the same analytical run (Figure 4). The LDIR data followed the previously discussed trend, with lower numbers of particles in the water when the spillway was open compared to when it was closed. The data also showed a general increase in the overall number of MPs at sites further to the west nearer the mouth of the Mississippi River, a major source of plastic pollution (Table 3). However, given the limited number of samples that were analyzed by LDIR, we hesitate to make a detailed comparison with fluorescence microscopy. Nevertheless, it is worth noting that, while the absolute numbers of particles detected by the two techniques were in the same magnitude (usually between a hundred and a thousand particles), LDIR tended to detect more particles. It should be emphasized that, unlike fluorescence microscopy that involves non-targeted staining of particles and assumes fluorescing particles are plastic, LDIR produces spectra of individual particles, confirming their identity. Moreover, regardless of the technique used to identify particles, some particles remain unidentified; these could be mixtures of polymers or polymers with adhering particles or biofilms, which complicate the spectra and decrease the probability of a library match. Thus, the fluorescence counts reported herein can be considered conservative.

Overall, we show that the Agilent’s LDIR analyzer is a powerful new automated analytical tool to rapidly detect and characterize MP pollution. A detailed comparison between LDIR and other chemical imaging techniques used for microplastic analyses will be the subject of a future report.

## Figures and Tables

**Table 1:** Geographic coordinates for sites where water was collected along the Mississippi Gulf Coast for microplastic analyses. Sites are listed west to east, with site numbers depicted in Figure 1.

Site #	Site Name	Reef Site	GPS Coordinates		Depth (m)	Sampling Dates	
			Lat.	Lon.		Open Spillway	Closed Spillway
1	St. Joe’s Pass	No	30.1068	−89.5528	3.7	July (2019)	January (2020)
2	Waveland Reef	Yes	30.2730	−89.3702	2.6	April (2019)	September (2019)
3	Bay St. Louis	No	30.3510	−89.3547	1.3	April (2019)	September (2019)
4	St. Stanislaus Reef	Yes	30.3023	−89.3272	1.9	April (2019)	September (2019)
5	TNC Bay St Louis	No	30.3451	−89.2949	1.5	April (2019)	September (2019)
6	Henderson Pt.	Yes	30.2926	−89.2711	3.0	April (2019)	September (2019)
7	Pass Christian	No	30.2850	−89.2371	3.8	July (2019)	January (2020)
8	Kittiwake Reef	Yes	30.3324	−89.1652	2.3	April (2019)	September (2019)
9	Biloxi Bay	No	30.3753	−88.8306	1.4	July (2019)	January (2020)
10	Middle Bay	No	30.3749	−88.3992	1.2	July (2019)	January (2020)

**Table 2:** Concentrations of microplastics (MPs, >~25 µm–5 mm) along the Mississippi Gulf Coast during freshwater inflows in the summer and fall of 2019 and winter of 2020 ^a^.

Site #	Location/Name	Open Spillway				Closed Spillway			
		*n*	Mean (Range) (MPs/L)	SD	Salinity (ppth) ^b^	*n*	Mean (Range) (MPs/L)	SD	Salinity (ppth) ^b^
1	St. Joe’s Pass	2	196 (12–381)	NA	0.39	1	309	NA	NA
2	Waveland Reef	5	73 (55–153)	50	0.32	2	174 (39–309)	NA	16.4
3	Bay St. Louis	4	49 (18–80)	25	0.67	3	50 (0–116)	60	NA
4	St. Stanislaus Reef	4	36 (18–65)	20	0.40	5	121 (34–99)	76	16.8
5	TNC Bay St. Louis	2	69 (38–100)	NA	0.54	3	39 (15–73)	30	15.3
6	Henderson Pt. Reef	3	30 (20–50)	17	3.14	5	139 (20–198)	76	17.2
7	Pass Christian	12	178 (55–328)	223	8.1	3	176 (124–202)	44	4.7
8	Kittiwake Reef	4	121 (22–196)	82	4.21	-	NA	NA	18.9
9	Biloxi Bay	8	179 (64–278)	82	13.5	3	94 (55–116)	34	9.3
10	Middle Bay	6	192 (39–326)	107	19	3	141 (41–263)	112	17

^a^ Data are blank-subtracted; NA = not available. ^b^ ppth = part per thousand; oysters require at least 8 ppth salinity to grow (Virginia Dept. of Environmental Quality).

**Table 3:** Identification of microplastics in a subset of samples from the Mississippi Sound by LDIR.

Location (Site # in Figure 1)	Spillway	MP Counts	Most Abundant Plastics Identified (% of Total)						
			Poly- ester	Acrylates/PU	Poly-amide	PP	PE	PA	PTFE
St. Joe’s Pass (1)	closed	1154	44.3	17.2	10.1	9.0	2.5	7.7	2.3
Pass Christian (7)	open	1061	20.4	42.3	14.4	7.8	5.2	0.9	0.8
Biloxi Bay (9)	closed	383	76.2	10.3	1.9	0.9	0.7	5.3	2.8
Middle Bay (10)	open	181	31.7	14.5	7.9	8.4	2.2	24.2	3.5
Middle Bay (10)	closed	76	36.1	30.3	6.6	1.6	5.7	3.3	6.6

PU = polyurethanes; PP = polypropylene; PE = polyethylene; PA = polyacetal; PTFE = polytetrafluoroethylene.
